# Bone marrow mammaglobin-1 (SCGB2A2) immunohistochemistry expression as a breast cancer specific marker for early detection of bone marrow micrometastases

**DOI:** 10.1038/s41598-020-70012-2

**Published:** 2020-08-03

**Authors:** Iman Mamdouh Talaat, Mahmood Yaseen Hachim, Ibrahim Yaseen Hachim, Ramez Abd El-Razak Ibrahim, Mohamed Abd El Rahman Ahmed, Hanan Yehia Tayel

**Affiliations:** 1 Clinical Sciences Department, College of Medicine, University of Sharjah, P.O. Box: 27272, Sharjah, UAE; 2 Sharjah Institute for Medical Research, University of Sharjah, Sharjah, UAE; 3 Department of Pathology, Faculty of Medicine, Alexandria University, Alexandria, Egypt; 4 College of Medicine, Mohammed bin Rashid University of Medicine and Health Sciences, Dubai, UAE; 5 Pathology Department, Military Medical Academy, Alexandria Armed Forces Hospital, Alexandria, Egypt; 6 Clinical Pathology Department, Military Medical Academy, Alexandria Armed Forces Hospital, Alexandria, Egypt

**Keywords:** Cancer, Breast cancer

## Abstract

Despite all the advances in the management of breast cancer (BC), patients with distance metastasis are still considered incurable with poor prognosis. For that reason, early detection of the metastatic lesions is crucial to improve patients’ life span as well as quality of life. Many markers were proposed to be used as biomarkers for metastatic BC lesions, however many of them lack organ specificity. This highlights the need for novel markers that are more specific in detecting disseminated BC lesions. Here, we investigated mammaglobin-1 expression as a potential and specific marker for metastatic BC lesions using our patient cohort consisting of 30 newly diagnosed BC patients. For all patients, bone marrow (BM) aspiration, BM biopsy stained by H&E and BM immunohistochemically stained for mammaglobin-1 were performed. In addition, the CA15-3 in both serum and bone marrow plasma was also evaluated for each patient. Indeed, mammaglobin-1 immuno-staining was able to detect BM micrometastases in 16/30 patients (53.3%) compared to only 5/30 patients (16.7%) in BM biopsy stained by H&E and no cases detected by BM aspirate (0%). In addition, our results showed a trend of association between mammaglobin-1 immunoreactivity and the serum and BM plasma CA15-3. Further validation was done using large publicly available databases. Our results showed that mammaglobin-1 gene expression to be specifically upregulated in BC patients’ samples compared to normal tissue as well as samples from other cancers. Moreover, our findings also showed mammaglobin-1 expression to be a marker of tumour progression presented as lymph nodes involvement and distant metastasis. These results provide an initial evidence for the use of mammaglobin-1 (SCGB2A2) immunostaining in bone marrow as a tool to investigate early BM micrometastases in breast cancer.

## Introduction

Despite the progress in breast cancer (BC) management, 20–30% of early-stage BC patients will end up with recurrence and distance metastasis^1^. Those patients usually experience worse prognosis and shortened median survival^2^. Moreover, 90% of the mortalities related to BC were found to occur in patients who presented with metastatic disease^3,4^.

For that reason, early detection of disseminated tumour cells (DTCs) and metastatic lesions, in addition to their effective management might be essential to reduce mortality and improve the patients’ outcome^5^. The discovery of novel markers for early detection of metastasis as well as the early intervention and monitoring for BC patients might represent a key factor in the management of such patients.

Many markers were proposed for the early detection of metastatic BC, however many of them showed low sensitivity and specificity, which limit their clinical utility^6^. This lack of sensitivity and specificity highlights the need for new and novel markers that can early detect metastasis and are BC specific.

Bone marrow (BM) is considered as one of the main sites for BC metastasis, for that reason, efforts were made to early detect malignant infiltrates in BM^7^. Cytokeratins including (CK19 & CK20) are considered as one of the most widely accepted protein markers for the detection of epithelial tumour cells in bone marrow^8,9^. Moreover, the serum levels of CA15-3 (MUC-1), which is a mucinous glycoprotein, is another commonly used tumor marker for preclinical detection of tumour recurrence and metastasis, in addition to patients’ follow up^10^. One of the limitations of the use of CA15-3 as a specific marker for BC metastasis is the fact that it is overexpressed in a group of other epithelial cancers including ovary, colon and lung^11^.

Previous reports also identified mammaglobin-1 (SCGB2A2), which is a glycoprotein that belongs to a larger family called uteroglobin^12^, as a potential diagnostic and prognostic marker for BC.

Indeed, 70–80% of the primary and metastatic BC biopsies were found to show evidence of mammaglobin-1 mRNA expression. Additionally, 80% of BC samples showed mammaglobin-1 protein expression by immunohistochemistry (IHC)^13^. While the diagnostic role of mammaglobin-1 expression in BC is well established, the utility of this marker as an early and tissue-specific marker of BC metastasis is yet to be illustrated^14^.

In this study, we used our patient cohort of 30 newly diagnosed BC patients with apparently non-metastatic primary lesion categorized as stage I, II and III who received no chemotherapy. All patients were investigated for bone marrow aspiration (BMA) and bone marrow biopsy (BMB) stained by H&E as well as BM immunohistochemical staining for mammaglobin-1. Besides, the serum and BM plasma CA15-3 were also evaluated for each patient and the correlation between the two markers was investigated. Due to the limited number of our patient cohort, different publicly available databases containing thousands of BC samples was also used to investigate the specificity of mammaglobin-1 as a marker of tumour progression and metastatic lesions in BC.

## Materials and methods

### Patients

Our cohort consisted of 30 BC patients with early stages (0-III). Those patients were selected from Alexandria Armed Forces Hospital during the period from March 2014 to December 2015. The clinicopathological data and medical records of all patients were retrieved and reviewed.

### Bone marrow biopsy

The BMB was obtained from the posterior superior iliac spine [PSIS] using the manual trephine needles. The obtained biopsy cores were fixed in 10% formol-saline for at least 24 h. The specimens then decalcified in formic acid-sodium citrate for 48 h.

Four µm thick sections were cut and then dehydrated in alcohol, cleared in xylene, embedded in paraffin wax, and stained with H&E stain.

### Bone marrow aspiration

The BMA was obtained from the same puncture just after obtaining the core through the same skin incision with the same needle but after being angled in a different direction. The aspirate was spread immediately on multiple glass slides and left to dry before being stained by Leishman stain.

### Immunohistochemical staining of BMB sections

Immunohistochemistry staining was performed on the BMB. In brief, the slides initially were incubated in peroxidase blocking solution for 10 min. The slides then incubated for 60 min with mammaglobin-1 monoclonal antibody available from DAKO for Anti-Mammaglobin, code no. M3625 diluted with DAKO Antibody Diluent code no. S 0809, pH 7.2–7.6, according to the recommended dilution (1:15–30). This is followed by applying labeled polymer to cover the section and incubate for 30 min. Then the whole section was covered by applying fresh prepared (DAB and chromogen) solution to be incubated afterwards for 5–10 min in the dark. The slides were counter-stained with hematoxylin stain and incubated for 15–20 min. Next, the slides were dehydrated by subsequent dipping into ascending grades of ethyl alcohol then cleared by two quick dips in xylene. Finally, the slides were mounted with DPX.

### Morphological interpretation

#### Bone marrow aspiration interpretation

Criteria of morphologically positive BM aspirate includes the presence of any large pleomorphic cells with hyperchromatic coarsely reticular nuclear chromatin, prominent or sharply defined nucleoli and moderately abundant variably basophilic cytoplasm ± vacuolation. These neoplastic cells occur either as tight small clumps or clusters ± individually dispersed cells. In addition, they are arranged either in "syncytial" formation or in Indian file pattern.

#### Bone marrow biopsy interpretation

In biopsy sections, the criteria of positive BM include:

1. The presence of neoplastic cells of same nuclear features as mentioned above, in randomly scattered small aggregates of 2–4 cells (forming micro-colonies) or in small but obvious sheet(s).
2. Frequently associated marrow stromal reaction in the form of one or more of the following:
3. Variable degrees of fibrous stromal response with formation of reticulin and/or collagen fibrosis among the involved areas with or without the formation of "desmoplasia".
4. Obvious interstitial increase in marrow eosinophils, histiocytes, plasma cells and/or fibroblasts.
5. Active "angiogenesis" or increased mean vascular density.
6. Increased osteoblastic/osteoclastic activities with the occurrence of trabecular bone erosion and/or appositional bone formation that lead to widening of BM trabeculae spaces.

#### Criteria of morphologically suspicious BM

In aspirate smears:

1. Absence of frank non-haemopoietic-looking neoplastic cells but rare or occasional presence of their single or clustered "bare" nuclei.
2. Associated increase in marrow osteoblasts and osteoclasts.

In biopsy sections:

1. Absence of obvious non-haemopoietic sheets or frank micro-metastatic colonies.
2. Presence of sticking one or more of the above-mentioned marrow stromal reactions either associated with morphologically uncertain micro-metastatic deposits or not.
3. The morphologic expectation of hidden metastatic cells that might be entangled among a prominent fibrotic reaction.

#### Criteria of morphologically negative BM

1. Morphological absence of any frank or suspicious individual cells, micro-metastatic colonies or sheets in all examined both marrow smears and sections.
2. Absence of any suspicious marrow stromal reactions in marrow sections.

#### Interpretation of immunohistochemistry staining

The cases were considered positive, if the cells showed diffuse brown coloration on cell membranes and/or in cytoplasm of cells.

#### CA15-3 serum and marrow plasma levels assessment

CA15‐3 serum and marrow plasma levels assessment were performed using the commercially available quantitative fully automated Abbott "Axsym" system (Abbot Laboratories, Abbot Park, IL).

From each patient, 150 µL of the sample were pipetted to one well of the reaction vessel where it was mixed with 115D8 MoAb‐coated microparticles forming an antigen–antibody complex. The reaction mixture was incubated, so the antigenic reactive determinants in the sample to bind to the antibody-coated micro-particles. An aliquot of the reaction mixture was transferred to the matrix cell where the micro-particles bound irreversibly to the glass fiber matrix. The matrix cell was washed to remove unbound materials. For the DF3 antibody, Alkaline Phosphatase conjugate was dispensed onto the matrix cell and bound to the Ag-Ab complex. The matrix cell was then washed to remove unbound materials. This is followed by adding the substrate to the matrix cell and the rate of fluorescent product formation was measured by the MEIA optical assembly.

### Data mining

Different large, publicly available databases were used to investigate the association between mammaglobin-1 expression levels and different clinicopathological data. This included ONCOMINE database, which contains data from 700 different datasets constituting samples from diverse cancers. In addition, we used GOBO database that includes 1881 BC patients’ samples to investigate the association between gene expression levels and the clinicopathological parameters. Moreover, the BC Gene-Expression Miner v4.0 database was also used to investigate the correlation between mammaglobin-1 (SCGB2A2) and CA15-3 (MUC-1) gene expression levels.

### Statistical analysis

Statistical analysis was performed using SPSS statistics software version 23. For our patient cohort, chi-square test was used to investigate the association between BMA, H&E stained BMB, mammaglobin-1 IHC and different clinicopathological parameters. The *p* value (< 0.05) was considered significant for all applied statistical tests. For the ONCOMINE database, the p-values was automatically calculated by the program using t-test. The figures were presented as box plots with median represented as the band inside the box, while the top and bottom of the boxes represent the 25th percentile and 75th percentile.

In breast cancer miner database as well as GOBO database, the association between gene expression levels and different clinicopathological parameters was done using Welch's test, in addition to Dunnett–Tukey–Kramer's tests.

### Ethics statement

The research was approved by the Research Ethics Committee of the Faculty of Medicine, Alexandria University, Egypt (approval number: 052808025). Biopsies from the patients were collected after informed consent according to the Helsinki declaration. All experiments and methods were performed in accordance with relevant guidelines and regulations.

## Results

### Bone marrow mammaglobin-1 immunoreactivity is more sensitive in detection of BM micrometastases than BMA and BMB H&E

To study the possible use of mammaglobin-1 as a marker for early prediction of BM metastasis in BC patients, we investigated the ability of mammaglobin-1 IHC in the detection of BM micrometastases compared to other classical markers and techniques using our patient cohort of 30 BC patients.

The presence or absence of BM micrometastases of all patients was determined by BMA and H&E stained BM trephine biopsy. The results were then compared to mammaglobin-1 BM IHC staining (Table 1).

**Table 1:** The distribution of BMA, H&E stained BMB and mammaglobin-1 IHC staining in our patient cohort.

Technique	No. of cases	%	*p*
**BMA**			0.0013*
Positive	0	0.0	
Negative	30	100.0	
**H&E stained BMB**			
Suspicious	5	16.7	
Negative	25	83.3	
**Mammaglobin-1 IHC staining**			
Positive	16	53.3	
Negative	14	46.7	

**Figure 1:**
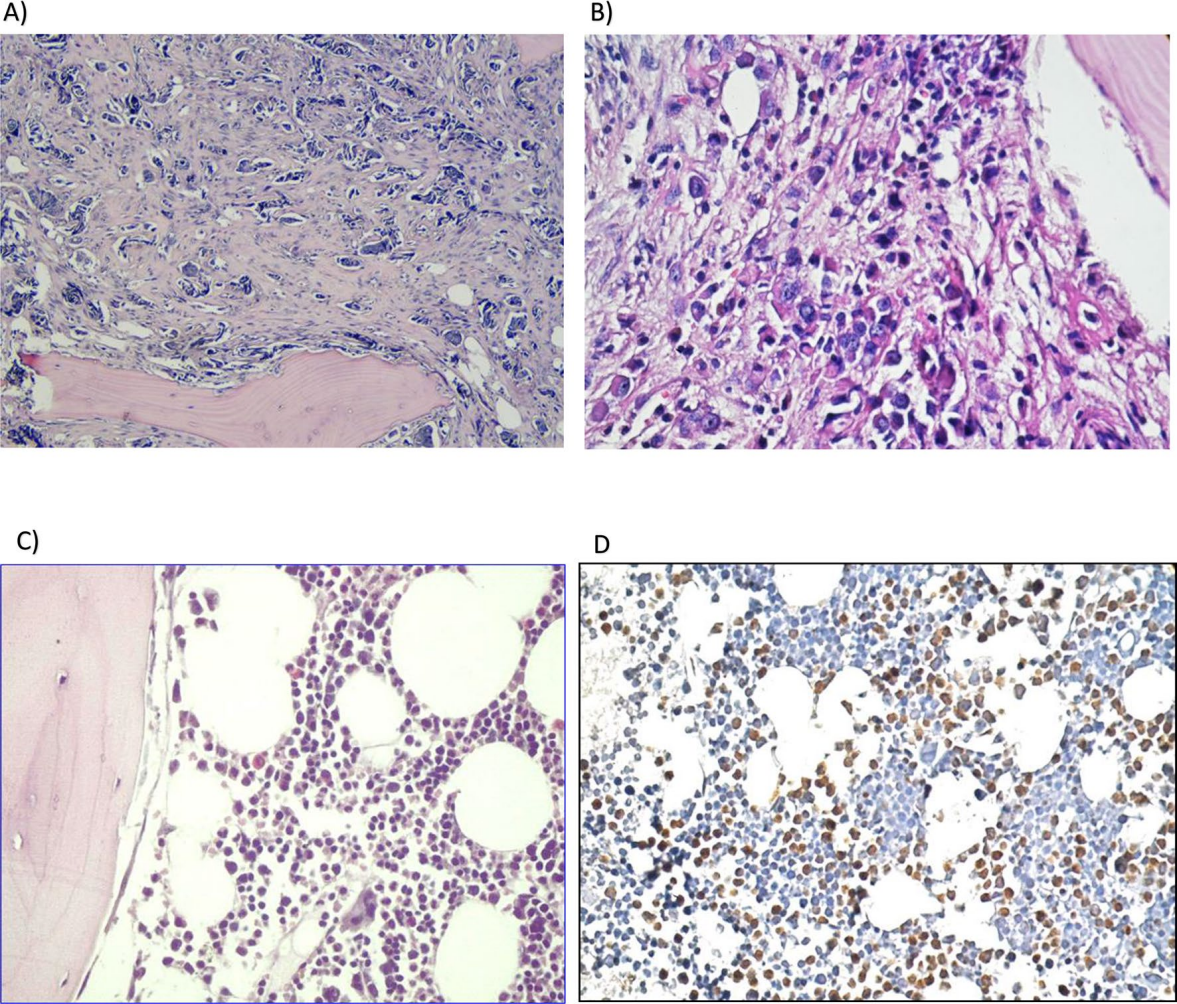
Representative pictures of the H&E and mammaglobin-1 IHC staining. (**A**) Positive case of metastatic invasive ductal carcinoma (IDC), the neoplastic epithelial cells forming nests in the fibrous stroma of the bone marrow (H&E, × 100). (**B**) Suspicious case of metastatic IDC shows hypercellularity and numerous inflammatory cells (plasma cells, histocytes and eosinophils) (H&E, × 400). (**C**) Suspicious case of metastatic breast carcinoma showing inflammatory infiltrates (plasma cells and eosinophils) (H&E, × 400). (**D**) The same case shown in C revealing brown cytoplasmic staining of mammaglobin-1 (positive stain) (Immunoperoxidase, × 400).

Bone marrow aspirate (BMA) was not able to detect any evidence of BM micrometastases in all 30 patients (0%). Moreover, H&E stained BMB showed suspected BM micrometastases in 5 patients (16.7%) presenting as hypercellularity, fibrosis, inflammatory infiltration (plasma cells, macrophage, and eosinophils), angiogenesis and osteoclastic hyperplasia (Figs. 1A–C). Mammaglobin-1 immunoreactivity, which is highly suggestive of BM micrometastases, was observed in 16 patients (53.3%) (*P* = 0.0013) (Fig. 1D).

Interestingly, 4/5 of the BMB suspicious cases (80%) showed positive IHC staining for mammaglobin-1 (Table 2). These results highlight mammaglobin-1 IHC staining as a possible sensitive and specific marker to detect BM micrometastases in BC samples compared to other classical methods.

**Table 2:** The association between mammaglobin-1 bone marrow immunoreactivity and different clinicopathological parameters.

	Mammaglobin-1 bone marrow immunoreactivity			*p*
	Positive	Negative	% of Positive	
**BMB H&E**				
Negative	13	12	52	*P* = 0.24
Positive	4	1	80	
**Histological type**				
Invasive ductal carcinoma	11	11	50	*P* = 0.54
Invasive lobular carcinoma	5	3	62.5	
**Stage**				
Stages 0,1	3	5	37.5	*P* = 0.29
Stages 2,3	13	9	59.09	
**LN status**				
LN negative	3	7	30	*P* = 0.09
LN positive	13	7	65	
Grade*				
Grade I	0	3	0	*P* = 0.019
Grade II	4	6	40	
Grade III	7	2	77	

*8 cases were lobular carcinoma, so they were not included in the grading system.

### Bone marrow mammaglobin-1 immunoreactivity is associated with more advanced disease and more aggressive phenotype

Next, we evaluated mammaglobin-1 positive cases and their association with classical clinicopathological parameters (Table 2). Despite the lack of statistical significance, our results showed that mammaglobin-1 positivity in the BM was doubled in more advanced stages (59.09%) (II & III) compared to the early stages (0 & I) (37.5%). Similarly, mammaglobin-1 immunoreactivity was more detected in LN positive samples (65%) compared to LN negative tumors (30%) (*P* = 0.09). Similarly, mammaglobin-1 expression was significantly higher in samples obtained from poorly differentiated tumors (77%) compared to no expression (0%) in samples obtained from well differentiated tumors (0%) (*P* = 0.019). This highlights that mammaglobin-1 positive BM expression to be associated with more advanced disease and more aggressive phenotype.

### Bone marrow mammaglobin-1 immunoreactivity and its association with serum and BM plasma CA15-3

CA15-3, is a mucinous glycoprotein and a product of mucin-1 (MUC-1) gene usually found in most of the epithelial cells^15^. The expression of CA15-3 was found to be overexpressed in many tumors including breast, ovarian, colon, lung, and other cancers^16,17^. Many reports showed that elevated serum CA15-3 is usually associated with more advanced disease and lymph node involvement^18,19^. Moreover, serial measurement of CA15-3 is commonly used for follow up and early detection of tumor recurrence and metastatic disease in BC patients^20,21^.

For that reason, we next evaluated the association between both serum and BM plasma CA15-3 levels and mammaglobin-1 BM immunoreactivity. The mean serum CA15-3 in our patient cohort was 19.79 ± 13.65 (ranged 3.7–61). The mean BM plasma CA15-3 level was 19.87 ± 13.78 (range 3.9–61.5). There was no statistically significant difference between serum and BM plasma CA15-3 levels (*P* > 0.05) (Table 3).

**Table 3:** Descriptive statistics for serum and BM plasma CA15-3.

	Serum CA15-3	BM plasma CA15-3
Range	3.7–61	3.9–61.5
Mean	19.79	19.87
S.D	13.65	13.78
T	0.98	
p	0.452	

Interestingly, our results showed that patients with positive BM mammaglobin-1 immunoreactivity had higher levels of serum CA15-3 (21.975 ± 17.852) compared to 17.292 ± 5.955 in the mammaglobin-1 IHC negative group. Similarly, BM plasma CA15-3 was also higher in the positive BM mammaglobin-1 IHC cases (21.925 ± 18.109) compared with mammaglobin-1 IHC negative cases (17.51 ± 5.8228) (Table 4). Those results further highlight the role of BM mammaglobin-1 IHC detection as a possible marker for the early detection of bone marrow micrometastases.

**Table 4:** Relation between serum and BM plasma CA15-3 and mammaglobin-1 IHC.

	N	Mean	S.D	Min	Max	t-test	*p*
**Serum CA15-3**							
Mammaglobin-1 IHC negative	14	17.2929	5.95502	7.60	27.80	0.874	0.358
Mammaglobin-1 IHC positive	16	21.9750	17.85208	3.70	61.00		
**BM plasma CA15-3**							
Mammaglobin-1 IHC negative	14	17.5143	5.82288	7.80	27.80	0.759	0.391
Mammaglobin-1 IHC positive	16	21.9250	18.10972	3.90	61.50		

### Mammaglobin-1 protein and RNA levels are highly expressed in breast tissue samples compared to other tissues

Our previous results showed mammaglobin-1 as a possible marker for early detection of bone marrow micrometastases in BC patients, for that reason, next we tried to further investigate its organ specificity in normal as well as malignant lesions from different tissues extracted from the human protein atlas , which is publicly available database.

**Figure 2:**
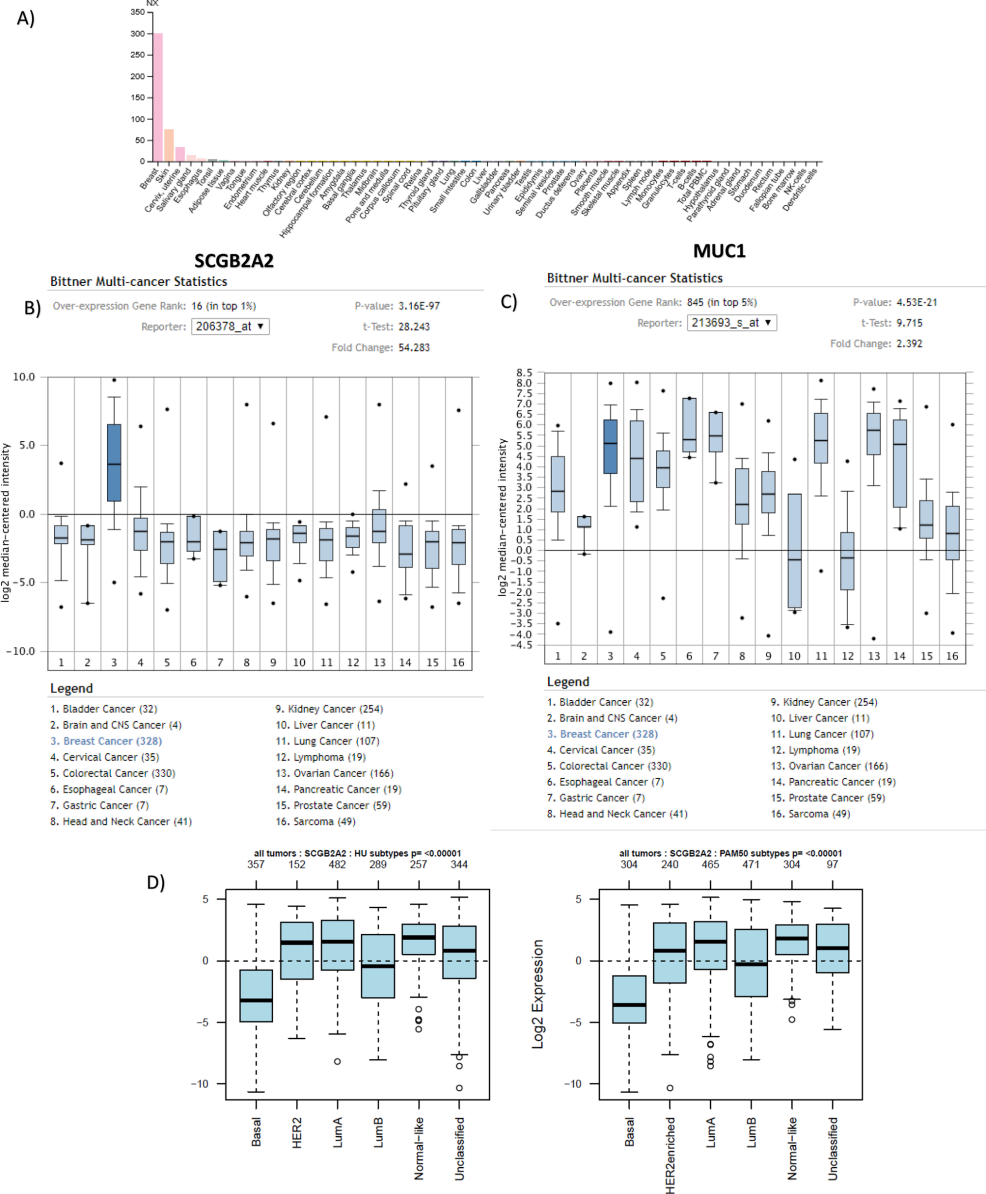
Mammaglobin-1 (SCGB2A2) expression in different normal as well as malignant tissues. (**A**) SCGB2A2 gene expression levels in different healthy body tissue using human protein atlas database. (**B**) SCGB2A2 gene expression levels in 1,500 cancer cases from different organs using Bittner multicancer dataset from the ONCOMINE database. (**C**) MUC1 gene expression levels in 1,500 cancer cases from different organs using Bittner multicancer dataset from the ONCOMINE database. (**D**) SCGB2A2 gene expression and its association with breast cancer subtypes according to Hu and PAM50 classification using 1881 breast cancer cases of GOBO database.

In healthy tissues, mammaglobin-1 mRNA levels showed highest expression in breast tissue, followed by skin, uterine cervix and salivary glands (Fig. 2A). Similarly, between malignant tissues, mammaglobin-1 expression was significantly higher in BC samples compared to all other cancer types (*P* = 3.16E−97) (Fig. 2B).

### Mammaglobin-1 gene expression levels showed higher tissue specificity in detecting BC tumors compared to CA 15-3

Next, and using the same in silico approach, we investigated the specificity of mammaglobin-1 compared to CA15-3, which is widely used marker for BC patients’ follow-up. Interestingly, our results showed that CA15-3 levels to be elevated in group of epithelial cancers with no specificity for BC. In comparison, mammaglobin-1 was specifically upregulated in BC samples compared to other tumors (Fig. 2B,C). This clearly demonstrates that mammaglobin-1 is a suitable candidate as a specific biomarker for breast lesions and is more specific than CA15-3.

### Mammaglobin-1 gene expression is associated with luminal A BC subtype and least expressed in the basal-like BC subtype

For further evaluation of the role of mammaglobin-1 in BC, we investigated its expression in different breast cancer subtypes using another database that includes 1800 BC samples. Indeed, we found mammaglobin-1 gene expression to be significantly correlated with luminal A breast cancer subtype and to be least expressed in the basal-like BC subtype (Fig. 2D).

### Mammaglobin-1 gene expression is upregulated in the more advanced BC disease

**Figure 3:**
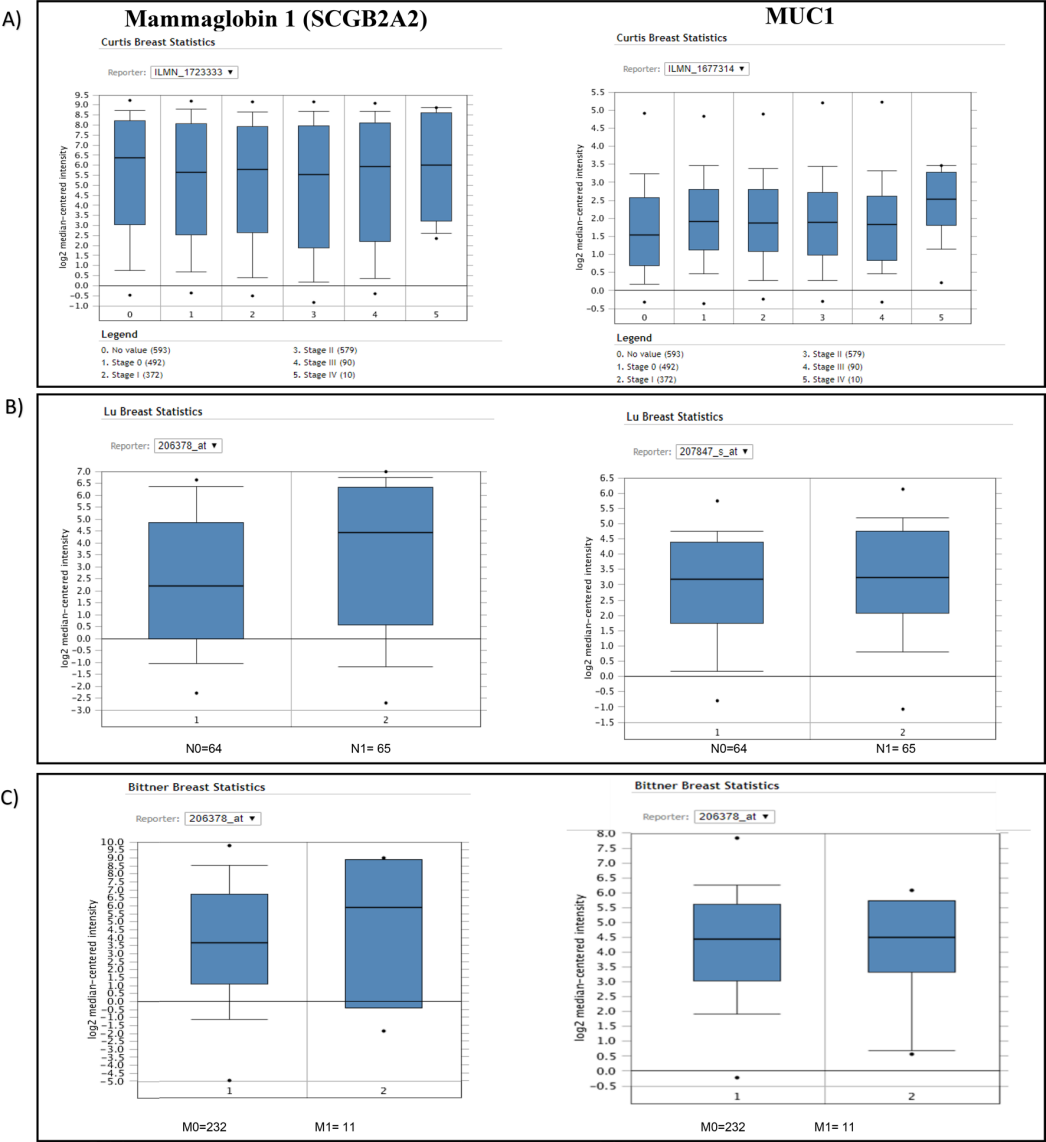
Mammaglobin-1 (SCGB2A2) gene expression in association with tumor progression. (**A**) Mammaglobin-1 and MUC1 gene expression and their association with tumor stage using Curtis dataset of ONCOMINE database. (**B**) Mammaglobin-1 gene expression and MUC1 its association with LN involvement using Lu dataset of ONCOMINE database. (**C**) Mammaglobin-1 gene expression and MUC1 its association with presence or absence of distant metastasis using Brittner dataset of ONCOMINE database.

Then, we evaluated the expression levels of mammaglobin-1 gene and its association with tumor progression. Our results showed that mammaglobin-1 expression to be steadily upregulated in the more advanced stages (stages III and IV) compared to the early stages (stages 0, I and II). Similarly, CA 15-3 also showed the same trend of overexpression in the more advanced stages (III & IV) compared to the early stages (I & II) (Fig. 3A).

Next, we investigated the association between mammaglobin-1 and LN involvement as well as distant metastasis, which are considered an important indicator of tumor progression. Our results clearly demonstrated an upregulation of the mammaglobin-1 gene expression in BC patients with LN positivity compared to LN negative tumors (Fig. 3B). Similarly, our results also showed that mammaglobin-1 gene expression to be overexpressed in patients with tumor metastasis compared to patients with no evidence of metastasis (Fig. 3C).

Interestingly, mammaglobin-1 showed higher power in differentiating LN status compared to CA15-3 (Fig. 3B). Similarly, mammaglobin-1 was also more able to differentiate metastatic samples from non-metastatic samples when compared to CA15-3 (Fig. 3C).

### Mammaglobin-1 gene expression levels is associated with higher incidence of bone metastasis compared to other metastatic sites

**Figure 4:**
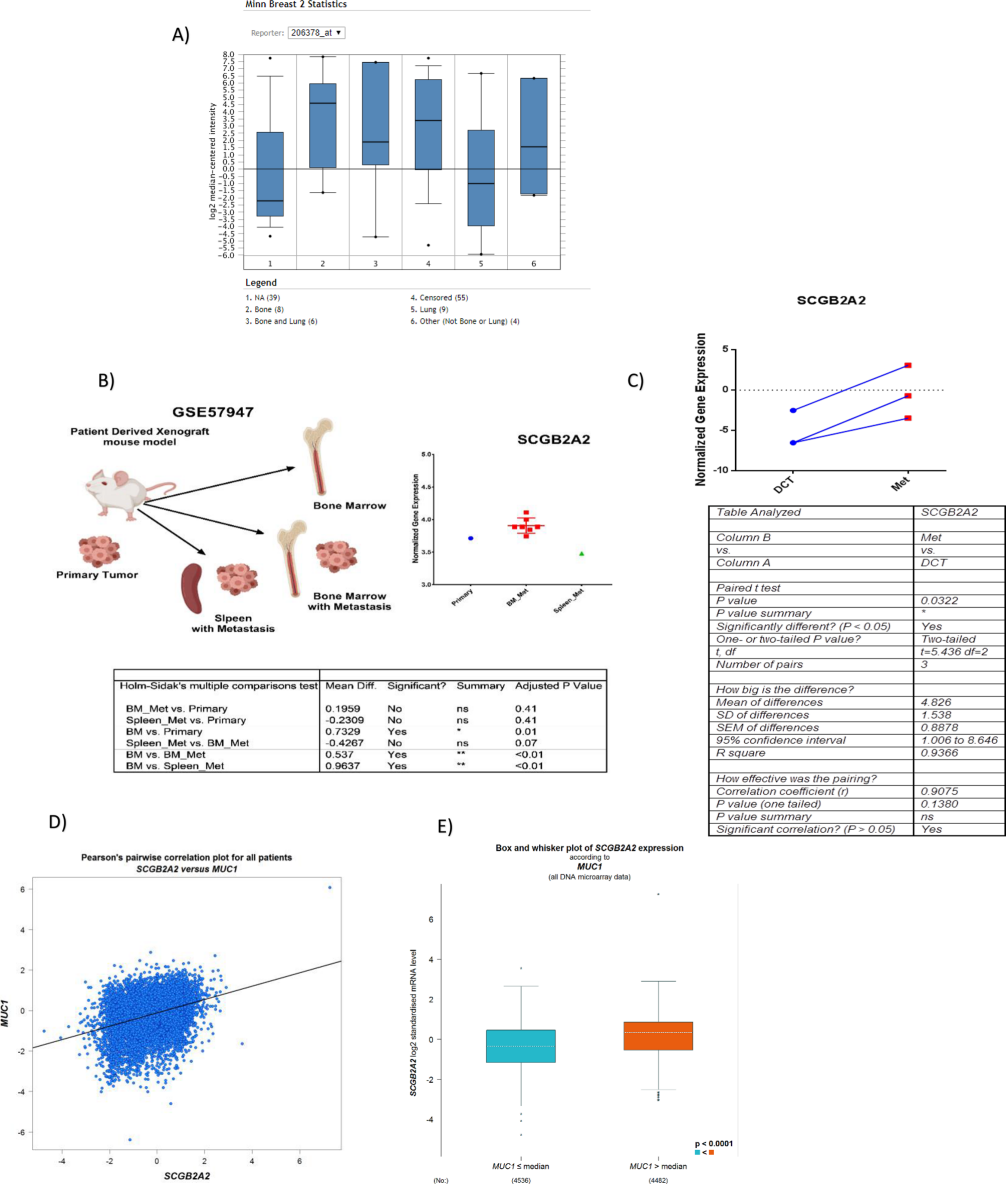
Mammaglobin-1 (SCGB2A2) gene expression in disseminated tumour cells (DTCs) compared to metastatic tumour cells (MTCs). (**A**) SCGB2A2 gene expression according to different metastatic sites in breast cancer using Minn dataset of ONCOMINE database. (**B**)The gene expression levels of SCGB2A2 in patients’ derived xenograft (PDX) of mice with BM metastasis compared to primary tumors using (GSE57947) dataset. (**C**)The gene expression levels of SCGB2A2 in DTCs compared to MTCs obtained from (GSE14776). (**D**)The Pearson’s correlation plot between SCGB2A2 and MUC1 gene expression levels in BC samples using BC Gene-Expression Miner v4.0 database. (**E**) The expression levels of SCGB2A2 in BC samples stratified according to MUC1 gene expression levels using BC Gene-Expression Miner v4.0 database.

Next, we investigated the association between mammaglobin-1 expression levels and different metastatic sites. Our results showed mammaglobin-1 to be higher in cases with bone metastasis compared to lung and other metastatic sites (Fig. 4A). These results further indicate the strong association between mammaglobin-1 expression and bone involvement in BC.

### Mammaglobin-1 expression can detect early disseminated tumor cells in BM

Then, we tried to investigate the role of mammaglobin-1 as an early marker for bone marrow metastasis. To achieve this, we extracted data profile from (GSE57947) dataset that represents a Patient Derived Xenograft (PDX) model established to identify genes that are associated with BM micro metastatic disease in breast cancer. Using our own in-house filtration method, we compared the gene expression levels of mammaglobin-1 in mice with primary tumors only compared to mice with BM metastasis as well as with splenic metastasis.

Even though the difference was not significant due to the limited number of mice, our results clearly showed higher levels of mammaglobin-1 in mice with BM metastasis compared to the primary tumor. Similarly, mammaglobin-1 levels were also high in mice with BM metastasis compared with mice with splenic metastasis (Fig. 4B).

### Mammaglobin-1 is progressively increased from early disseminated tumor cells to overt bone metastasis

Disseminated tumor cells (DTCs) in the BM of BC patients are commonly identified in early stage of the disease, however, their potential to initiate metastases is not known. Indeed, genes that are essential to develop bone metastasis might represent the ideal marker for micro-metastasis prediction. For that reason and using the same bioinformatic approach, we investigated the expression levels of mammaglobin-1 in DTCs from BM aspirates and then we compared them to matching overt bone metastasis obtained from CT guided biopsies of bone metastases of BC patients.

Our results showed that mammaglobin-1 expression was significantly upregulated in the overt metastatic tumour cells (MTCs) compared to only DTCs (*P* = 0.03) (Fig. 4C). These results indicate that mammaglobin-1 might be essential for DTCs to progress into overt MTCs and play role in facilitating their progression.

### Mammaglobin-1 gene expression is highly associated with CA15-3 gene expression in large BC patient cohort

Due to the limited number of our patient cohort and to have better idea about the association between mammaglobin-1 expression and CA15-3 levels, we investigated the association between mammaglobin-1 gene expression and CA15-3 gene expression levels in 9,000 BC samples obtained from publicly available databases.

Indeed, our results showed a strong positive correlation between CA15-3 expression and mammaglobin-1 expression (r = 0.34, *P* < 0.0001). Moreover, splitting BC samples according to CA15-3 expression showed that BC samples that had CA15-3 expression more than the median have a significant upregulation in the mammaglobin-1 gene expression. This further confirms the strong association between mammaglobin-1 expression and CA15-3 in BC patients’ samples (Figs. 4D,E) and highlights the possible use of mammaglobin-1 as an alternative marker for CA15-3 in the prediction of BM micrometastases in BC patients.

## Discussion

The prognosis of BC patients was found to be dramatically influenced by presence or absence of metastatic lesions. While the 5-year survival of patients with localized disease reaching up to 99%^22^, this rate dramatically decline to only 25% in patient presented with metastatic lesions^22,23^. This includes the presence of occult micrometastases to BM that was found to be present in around 30% of patients with early stage breast cancer and found to be related with worse patient’s outcome^24^.

For that reason, great efforts has been made during the last decades for the discovery of metastasis-related biomarkers that can be used for the prediction and early detection of disseminated tumor cells and metastatic lesions in BC^25–27^.

Known the fact that an ideal marker should specifically and universally be expressed on all BC cells. In addition, it should also be easy to detect and has clinical relevance, number of single and multi-marker assays were evaluated^28^.

Unfortunately, the lack of solely tissue specific marker for metastatic BC lesions is still considered as one of the major challenges in that field^29^. For examples, CA15-3, which is widely used as biomarker for BC patients’ follow-up and progression, was found to have low BC specificity^28^. Another major challenge, is the lack of international consensus and protocols for the various immunocytochemical and molecular markers used to detect occult micrometastases in the BM of BC patients^29^.

For that reason, here we investigated the possible use of BM mammaglobin-1 expression as a possible and single marker of early detection of BM micrometastases in BC patients.

Our results using our patient cohort revealed that BM IHC staining for mammaglobin-1 was more sensitive in the detection of early BM micrometastases compared to other conventional techniques including BMA and BMB stained by H&E. Indeed, this goes with the fact that using conventional H&E stain for detection of early metastatic lesions might not be able to detect very small amounts of disseminated occult tumor cells in different metastatic sites including lymph nodes^28^.

Additionally, our results showed a trend of association between mammaglobin-1immunoreactivity and the serum and BM plasma CA15-3, which is the most widely used serum marker for initial work-up for BC diagnosis, besides to its use as early marker for recurrence and/or metastatic breast disease^20,21,30^.

Due to the limited number of our patient cohort, different publicly available databases that contain thousands of BC samples were also used to investigate the specificity of mammaglobin-1 as a marker of tumour progression and metastatic lesions in BC.

Our results clearly demonstrated that mammaglobin-1 gene expression is specifically upregulated in BC patients compared to CA15-3, which showed upregulation in a group of other epithelial cancers including esophageal, gastric, lung and ovary. The specificity of mammaglobin-1 to BC was previously highlighted in a group of reports. Indeed, mammaglobin-1 expression was found to be strictly confined to normal mammary tissue as well as BC tissues^31,32^.

Moreover, our findings that mammaglobin-1 expression to be upregulated in the more advanced BC stages; with LN positive and/or with metastatic lesions also go with previous reports that showed human mammaglobin expression to be more evident in the advanced stages of BC compared to earlier stages^33^.

The sensitivity of mammaglobin-1 BM immunoreactivity to detect bone marrow micrometastases compared to BMA and BMB also goes with previous reports that showed mammaglobin expression as a sensitive marker for micrometastases in other tissues like sentinel lymph nodes^34–40^.

The superiority of mammaglobin-1 in differentiating LN positive from LN negative tumors as well as metastatic from non-metastatic lesions compared to CA15-3 clearly highlights the possible use of BM mammaglobin-1 immunoreactivity as well as gene expression levels as an ideal marker for early detection of BM micrometastases in BC.

One of the main limitations of our study is the small samples size of our cohort, in addition to the lack of the patients’ follow-up data in that cohort. This limitation is not restricted to our cohort, but also appear in many reports that investigated the clinical utility of different prognostic and predictive markers in BC. However, and to overcome this limitation, we tried to use additional bioinformatic approach to further validate our data. The main advantage of this approach is the ability to investigate gene expression levels as well as protein levels of mammaglobin-1 (SCGB2A2) in large patients’ cohort reaching to thousands of patients and compare it with a wide range of clinicopathological parameters including patient’s outcome.

In conclusion, our results provide an initial evidence for the use of mammaglobin-1 (SCGB2A2) IHC expression in BM as a tool to investigate early BM micrometastases in BC. Despite the limited number of cases, our data clearly showed mammaglobin-1 IHC expression as a sensitive and specific tool to detect occult BM micrometastases in a relatively early-stage disease. Indeed, those results need to be validated in larger patient cohort to address their clinical utility.

## Abbreviations

BC: Breast cancer
BM: Bone marrow
BMA: Bone marrow aspiration
BMB: Bone marrow biopsy
DTCs: Disseminated tumor cells
IHC: Immunohistochemistry
